# Construction of a risk prediction model for lung infection after chemotherapy in lung cancer patients based on the machine learning algorithm

**DOI:** 10.3389/fonc.2024.1403392

**Published:** 2024-08-09

**Authors:** Tao Sun, Jun Liu, Houqin Yuan, Xin Li, Hui Yan

**Affiliations:** ^1^ Department of Hematology and Oncology Laboratory, The Central Hospital of Shaoyang, Shaoyang, Hunan, China; ^2^ Department of Scientific Research, The First Affiliated Hospital of Shaoyang University, Shaoyang, Hunan, China

**Keywords:** lung infection, chemotherapy, machine learning, logistic regression, predictive model, nomogram

## Abstract

**Purpose:**

The objective of this study was to create and validate a machine learning (ML)-based model for predicting the likelihood of lung infections following chemotherapy in patients with lung cancer.

**Methods:**

A retrospective study was conducted on a cohort of 502 lung cancer patients undergoing chemotherapy. Data on age, Body Mass Index (BMI), underlying disease, chemotherapy cycle, number of hospitalizations, and various blood test results were collected from medical records. We used the Synthetic Minority Oversampling Technique (SMOTE) to handle unbalanced data. Feature screening was performed using the Boruta algorithm and The Least Absolute Shrinkage and Selection Operator (LASSO). Subsequently, six ML algorithms, namely Logistic Regression (LR), Random Forest (RF), Gaussian Naive Bayes (GNB), Multi-layer Perceptron (MLP), Support Vector Machine (SVM), and K-Nearest Neighbors (KNN) were employed to train and develop an ML model using a 10-fold cross-validation methodology. The model’s performance was evaluated through various metrics, including the area under the receiver operating characteristic curve (ROC), accuracy, sensitivity, specificity, F1 score, calibration curve, decision curves, clinical impact curve, and confusion matrix. In addition, model interpretation was performed by the Shapley Additive Explanations (SHAP) analysis to clarify the importance of each feature of the model and its decision basis. Finally, we constructed nomograms to make the predictive model results more readable.

**Results:**

The integration of Boruta and LASSO methodologies identified Gender, Smoke, Drink, Chemotherapy cycles, pleural effusion (PE), Neutrophil-lymphocyte count ratio (NLR), Neutrophil-monocyte count ratio (NMR), Lymphocytes (LYM) and Neutrophil (NEUT) as significant predictors. The LR model demonstrated superior performance compared to alternative ML algorithms, achieving an accuracy of 81.80%, a sensitivity of 81.1%, a specificity of 82.5%, an F1 score of 81.6%, and an AUC of 0.888(95%CI(0.863-0.911)). Furthermore, the SHAP method identified Chemotherapy cycles and Smoke as the primary decision factors influencing the ML model’s predictions. Finally, this study successfully constructed interactive nomograms and dynamic nomograms.

**Conclusion:**

The ML algorithm, combining demographic and clinical factors, accurately predicted post-chemotherapy lung infections in cancer patients. The LR model performed well, potentially improving early detection and treatment in clinical practice.

## 1 Introduction

Lung cancer, being one of the most prevalent malignant neoplasms globally, presents a substantial risk to both the survival and well-being of affected individuals (1). The World Health Organization’s data indicates that lung cancer exhibits the highest incidence and mortality rates among all cancer types (2). Despite notable advancements in lung cancer therapy, the effective management of post-chemotherapy complications remains a significant hurdle (3–5). Of particular concern is the high prevalence of lung infections following chemotherapy in lung cancer patients, which seriously affects the therapeutic effect and survival quality of patients (6). The presence of lung infections in lung cancer patients not only exacerbates their health status but also has the potential to impede or halt chemotherapy, thereby impacting the overall efficacy of treatment. Furthermore, lung infections contribute to escalated medical expenses, extended hospital stays, and heightened mortality rates (7). Consequently, the timely and precise identification of the likelihood of lung infections following chemotherapy is crucial for informing clinical interventions and enhancing patient outcomes.

The utilization of ML technology in the healthcare sector has experienced significant growth in recent years, showcasing robust data processing and pattern recognition capabilities. ML algorithms have exhibited promise and efficacy in lung cancer diagnosis, treatment selection, and prognosis assessment (8, 9). Notably, the analysis of extensive clinical data through ML algorithms can aid healthcare professionals in identifying potential disease development patterns, facilitating personalized treatment strategies, and enhancing treatment outcomes (10–12). Conventional approaches to evaluating the risk of lung infection rely heavily on the subjective judgment and clinical expertise of healthcare professionals, necessitating a greater degree of objectivity and precision. In light of this prevailing situation, the utilization of ML technology presents novel opportunities for addressing this issue by leveraging ML algorithms to analyze extensive patient data, potential correlations and patterns can be identified, enabling healthcare providers to make more precise predictions regarding the likelihood of lung infection following chemotherapy in individuals with lung cancer.

In recent studies, researchers have utilized various ML algorithms to create predictive models aimed at aiding physicians in evaluating the likelihood of complications in lung cancer patients following chemotherapy or surgical procedures. While previous research has explored the application of ML in forecasting complications in lung cancer patients, there is a notable scarcity of studies focusing on predicting the likelihood of lung infection following chemotherapy. Consequently, the current study seeks to address this gap by introducing and refining a prediction model utilizing ML algorithms to identify lung cancer patients at risk of post-chemotherapy lung infection. This study posits that an interpretable ML-based algorithm will achieve the most accurate predictions if significant predictors are identified through an effective feature selection method. Therefore, the objective of this study was to create and evaluate a proficient and interpretable ML system for forecasting the likelihood of lung infection following chemotherapy in Chinese lung cancer patients. Our research findings offer a novel approach for early identification of infection risk in lung cancer patients while also contributing to the advancement of ML in oncology clinical investigations. Moving forward, we intend to enhance the precision and reliability of the model, facilitate its integration into clinical settings, and offer enhanced scientific and precise assistance for the care and oversight of lung cancer patients.

## 2 Materials and methods

### 2.1 Study design

**Figure 1:**
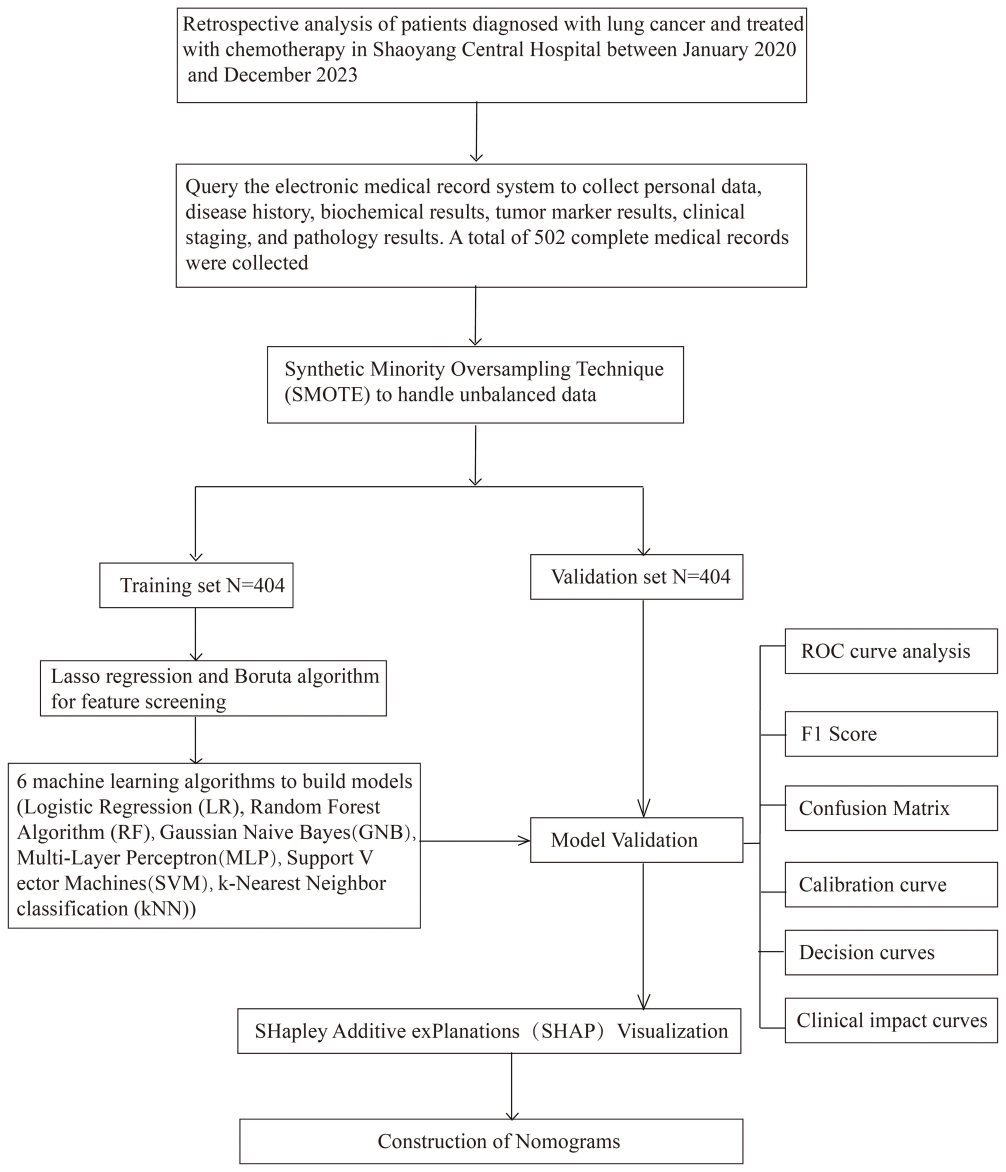
Research flowchart.

This study was conducted to develop a machine learning-based model for predicting the risk of lung infections following chemotherapy in lung cancer patients. The retrospective study included a cohort of 502 lung cancer patients who had undergone chemotherapy, aged 18 years and above, and had completed at least one cycle of treatment. Data encompassing demographic details, medical history, chemotherapy specifics, and blood test results were extracted from the hospital’s electronic medical record system. The SMOTE algorithm is used to solve the category imbalance problem. The Boruta algorithm and LASSO regression performed feature screening to identify the features most associated with the risk of lung infection. Subsequently, a range of ML models, including LR, RF, GNB, MLP, SVM, and KNN, were developed and refined by applying a 10-fold cross-validation methodology. The performance of these models was assessed using various metrics, including accuracy, sensitivity, specificity, positive predictive value, negative predictive value, F1 score, Kappa score, AUC, calibration curve, calibration curves, Clinical Impact Curve and confusion matrix. To enhance the transparency and interpretability of the model, the SHAP method was employed to interpret the predicted results and elucidate the impact of each feature on the predictions, thereby offering a practical reference for clinicians. **Figure 1** explains the overall workflow of the proposed system more clearly.

### 2.2 Study data

This retrospective study examined data from lung cancer patients at The Central Hospital of Shaoyang between January 2020 and December 2023. The study included adult patients aged 18 years and older who had not experienced lung infections within a week before receiving chemotherapy. Patient records with missing or abnormal data were excluded to maintain data quality. The study’s rigorous inclusion and exclusion criteria aimed to ensure the completeness and reliability of the information on included cases, thus providing a high-quality database for evaluating the risk of lung infections in lung cancer patients after chemotherapy. Inclusion criteria: (i) adult patients aged ≥18 years, (ii) patients diagnosed with lung cancer and treated with chemotherapy, (iii) patients who did not have any lung infection before chemotherapy, and (iv) patients with complete clinical information; Exclusion criteria: (i) patients with mental illness or intellectual disability, (ii) patients with missing or abnormal data, and (iii) exclusion of patients with a combination of other tumors.

### 2.3 Research variables

The study encompassed 36 predictors related to demographic factors (gender, age), lifestyle habits (history of alcohol consumption, history of smoking), medical history (history of diabetes, history of hypertension, history of coronary heart disease), physical characteristics (BMI), disease severity (stage at diagnosis, histologic features, presence or absence of pleural effusion), treatment information (cycles of chemotherapy, number of hospitalizations), and laboratory values (leukocytes, erythrocytes, hemoglobin, platelets, percentage of neutrophils, percentage of lymphocytes, percentage of monocytes, NLR, NMR, neutrophil-platelet count ratio (NPR), indirect bilirubin, alanine aminotransferase, glutamine aminotransferase, total bilirubin, direct bilirubin, total protein, albumin, globulin, white globule ratio, urea, creatinine, uric acid, and CEA). Of these, gender, age, history of alcohol consumption, history of smoking, history of diabetes mellitus, history of hypertension, history of coronary artery disease, BMI, tumor typing, cycles of chemotherapy, number of hospitalizations, and the presence or absence of pleural effusions were the data before the last chemotherapy session. The other laboratory data were obtained after the last chemotherapy. A brief description of the study variables is given in **Table 1**.

**Table 1:** Description of the study variables.

SN	Predictors	Description	Types	Values
1	Gender	Sex of the patient	Categorical	1 male 2 female
2	Age	Age of the patient (years)	Continuous	35-83
3	Drink	History of alcohol consumption	Categorical	0 No history of alcohol consumption 1 History of alcohol consumption
4	Smoke	History of smoking	Categorical	0 No history of smoking 1 History of smoking
5	Diabetes	History of diabetes	Categorical	0 No history of diabetes 1 History of diabetes
6	Hypertension	History of Hypertension	Categorical	0 No history of hypertension 1 History of hypertension
7	CHD	History of coronary heart disease	Categorical	0 No history of coronary heart disease 1 History of coronary heart disease
8	BMI	Body mass index (kg/m2)	Continuous	11.43-31.83
9	Stage	Stage at diagnosis, Count (%)	Categorical	Stage 1 24(4.78%) Stage 2 59(11.75%) Stage 3 204(40.64%) Stage 4 215(42.83%)
10	Histology	Histologic features, Count (%). 1, Adenocarcinoma; 2, Squamous; 3, SCLC; 4, Other lung cancers	Categorical	Grade1 222(44.22%) Grade2 189(37.65%) Grade3 80(15.94%) Grade4 11(2.19%)
11	Chemotherapy cycles	The Number of chemotherapy cycles	Continuous	1-32
12	Hospitalizations	Total number of hospitalizations	Continuous	1-45
13	PE	The presence of pleural effusion	Categorical	0 No pleural effusion 1 With pleural effusion
14	WBC	White blood cell	Continuous	1.31-60.80
15	RBC	Red blood cell	Continuous	1.44-6.20
16	HGB	Hemoglobin	Continuous	53.00-9792.00
17	PLT	Platelet	Continuous	22.00-631.00
18	NEUT	Percentage of Neutrophil	Continuous	0.44-98.21
19	LYM	Percentage of Lymphocytes	Continuous	1.42-65.50
20	NLR	Neutrophil-Lymphocyte count ratio	Continuous	0.02-69.16
21	NMR	Neutrophil-Monocyte count ratio	Continuous	0.01-311.37
22	NPR	Neutrophil-Platelet count ratio	Continuous	0.01-3.67
23	MONO	Percentage of Monocytes	Continuous	0.30-63.20
24	IBIL	Indirect bilirubin	Continuous	2.20-90.40
25	ALT	Glutamic pyruvic transaminase	Continuous	2.70-888.60
26	AST	Aspartate aminotransferase	Continuous	3.80-591.20
27	TBIL	Total bilirubin	Continuous	1.90-297.60
28	DBIL	Direct bilirubin	Continuous	0.13-207.20
29	TP	Total protein	Continuous	22.50-85.80
30	ALB	Albumin	Continuous	10.70-51.04
31	GLB	Globulin	Continuous	11.96-51.90
32	A/G	White ball ratio	Continuous	0.48-3.99
33	Urea	Urea	Continuous	1.25-32.97
34	CREA	Creatinine	Continuous	34.70-367.90
35	UA	Uric acid	Continuous	78.30-1201.40
36	CEA	CEA	Continuous	0.20-1500.00

### 2.4 Diagnostic criteria of pulmonary infection after chemotherapy

The diagnostic criteria for pulmonary infection in patients with lung cancer following chemotherapy encompass a body temperature exceeding 38°C, the presence of clinical symptoms indicative of pulmonary infection (e.g., cough and expectoration), the identification of moist rales in the lungs, and the visualization of a distinct infectious focus on CT imaging. Should a lung cancer patient meet at least three of these criteria within 14 days post-operation, a diagnosis of post-chemotherapy lung infection is warranted.

### 2.5 Feature screening

#### 2.5.1 Least absolute shrinkage and selection operator

The LASSO regression enhances model refinement by implementing a penalty function that compresses certain regression coefficients, thereby enforcing a constraint on the sum of their absolute values to be below a predetermined threshold (13, 14). We utilize the glmnet package in R for LASSO regression, setting family=“binomial” to apply to our binary outcome data. The key parameter alpha is set to 1, and the LASSO method is used entirely. Through cross-validation with the cv.glmnet function, we chose two lambda values: lambda.min and lambda.1se. The former minimizes the cross-validation error, while the latter provides a cleaner model, which together help us to balance the complexity of the model with the prediction accuracy. Ultimately, we filter out variables that are significant to the predictions based on non-zero coefficients, simplifying the model and improving its interpretability.

#### 2.5.2 Boruta

The Boruta algorithm is a Random Forest-based feature selection and packaging algorithm that evaluates the importance of features by generating “shadow variables” corresponding to each original variable in the dataset (15). In particular, Boruta (Version: 8.0.0) is executed to perform feature selection, where the algorithm iteratively compares the importance of each original variable with its shadow variable, and determines the importance of each variable over 500 iterations or until all variables are stable. Importance results are extracted with the attStats function and formatted with a customized adjustdata function (16).

### 2.6 Machine learning algorithms

#### 2.6.1 Logistic regression algorithm

In this study, we used a logistic regression (LR) model to predict the probability of infection in patients receiving chemotherapy, defined as a binary classification problem that predicts the risk of infection based only on clinical features (17). The logistic regression model used L2 regularization with the regularization factor (C) set to 1.0, a maximum number of iterations of 100, and a convergence tolerance (tol) of 0.0001.These parameters help prevent model overfitting while ensuring convergence and computational efficiency of the algorithm.

#### 2.6.2 Random forest algorithm

The RF algorithm is an ML technique that enhances predictive accuracy by generating multiple decision trees. RFs excel in analyzing extensive datasets with high-dimensional features, effectively managing intricate relationships among data variables (18). In this research, RFs are employed to identify non-linear associations and enhance the model’s ability to generalize. In the Random Forest model, the Gini Index is used as the splitting criterion, the number of trees is set to 20, the maximum depth of the tree is not restricted, and the minimum impurity reduction is set to 0.0. This parameter configuration is designed to allow the model to fully learn the complex structure in the data, and to improve the accuracy and generalization of the prediction.

#### 2.6.3 Gaussian Naive Bayes algorithm

The GNB classifier is a straightforward probabilistic model grounded in Bayes’ theorem, predicated on the feature independence assumption. While this assumption may not hold true in all practical scenarios, GNB remains highly effective in numerous instances owing to its simplicity and computational efficiency (19). The Gaussian Naive Bayes model does not set a specific prior probability, and the variable smoothing parameter is set to 1e-09. this setting allows the model to be more accurate when performing probability calculations, especially when dealing with datasets with continuous characteristics.

#### 2.6.4 Multi-layer perceptron algorithm

The MLP is a feed-forward artificial neural network model capable of processing data through multiple layers to learn non-linear features (20). It is well-suited for complex pattern recognition tasks. In this research, we employ MLP to develop a sophisticated predictive model for assessing the risk of lung infection following chemotherapy, the multilayer perceptron model uses ReLU as the activation function, and the structure of the hidden layer is set to two layers containing 20 and 10 neurons, respectively, with a maximum number of iterations of 20.

#### 2.6.5 Support vector machine algorithm

Support Vector Machine (SVM) is robust classifiers utilized to discern between classes by identifying optimal decision boundaries within data points. SVMs are especially adept at processing high-dimensional data and excel in scenarios where data boundaries are ambiguous (21, 22). In this study, the SVM model selects Radial Basis Function (RBF) as the kernel function, with the regularization parameter C set to 1.0 and the tolerance to 0.001. This setting helps the model to effectively identify complex decision boundaries while controlling overfitting when dealing with high-dimensional data.

#### 2.6.6 K-Nearest neighbor algorithm

The KNN is utilized to predict the category of a given sample point by examining the categories of its K-nearest neighbors. This method, known for its simplicity and intuitive nature, does not necessitate explicit model training (23). In this study, The number of neighbors of the KNN model is set to 5 and a uniform weighting method is used. This setting simplifies the computational process of the model and allows the model to predict the classification of new samples based directly on the nearest few samples for effective classification.

### 2.7 SHAP interpretability analysis

The SHAP is a technique utilized to interpret predictions generated by ML models, particularly those that are intricate and incorporate numerous features (24). The fundamental principle underlying this method involves the computation of the incremental impact of individual features on the model’s output, enabling interpretation of the model’s behavior at both a global and local scale. This is achieved through the development of an additive explanatory model that considers all features as contributors, thereby facilitating the calculation of the average incremental impact of each feature across all feasible feature combinations to derive a SHAP value for each feature, which provides both global and local interpretations, helping to understand which features are the main influences on model predictions, as well as the predictions of individual samples—factors, as well as the prediction results for a single sample (25).

### 2.8 Statistical analysis

All data analyses in this study were performed using SPSS (17.0), R language (version 4.3.2), Matlab (version R2021a), and Python (version 3.7). The initial analysis of the data set involved the application of descriptive statistics. Data points adhering to a normal distribution were represented as mean ± standard deviation, while those deviating from normal distribution were represented as median (quartiles). Subsequently, the independent samples t-test was employed to compare two groups with normally distributed data. In contrast, the Mann-Whitney U test was utilized to compare two groups with non-normally distributed data. For count data, frequencies and percentages were used to characterize group variances, while the chi-square test or Fisher’s exact probability method was employed to assess inter-group discrepancies. We solved the problem of sample imbalance by oversampling a small number of classes and thereby solving the sample imbalance problem through the SMOTE algorithm based on Matlab software. To construct the predictive model, the dataset was partitioned randomly into a training subset comprising 70% of the total data and a test subset comprising 30%. Subsequently, six ML algorithms were employed to train the model using the training subset data. During the model training process, a 10-fold cross-validation method is used to optimize the model parameters and prevent the occurrence of overfitting phenomenon. LASSO regression analysis was conducted utilizing the glmnet package [4.1.7] in R to analyze cleaned data and derive coefficient values of variables, logarithmic values of lambda, and regularized values of L1, followed by data visualization. The Boruta algorithm was implemented using Boruta 8.0.0 [4.1.7] in R. Interpretability analysis was carried out using the Python libraries shap=0.43.0. Statistical significance levels were established at *P*<0.05.

## 3 Results

### 3.1 Patient characteristics

This study assembled a cohort of 502 lung cancer patients who did not have lung infections before undergoing chemotherapy. The median age of the patients was 65 years (range: 58-71 years), with 404 (80.48%) being male and 98 (19.52%) being female. We used the SMOTE algorithm for data imbalance. The original data of 502 cases contained 404 non-infected cases, 98 infected cases, and 19.52% of infected cases, and the processed data of 808 cases contained 404 non-infected cases, 404 infected cases, and 50.00% of infected cases. A comparison of baseline characteristics between the two groups revealed statistically significant differences in chemotherapy cycles, hospitalizations, WBC, pulmonary embolism, Gender, CREA, Histology, alcohol consumption, smoke, CHD, NEUT, LYM, NMR, NPR, IBIL, TBIL, and NLR (*P* < 0.05), as shown in **Table 2**.

**Table 2:** Baseline characterization and comparison.

Variables	Total (n = 808)	Pulmonary infection after chemotherapy for lung cancer		*P*
		No (n = 404)	Yes (n = 404)	
Age	65.00 [59.00, 70.00]	65.00 [58.00, 71.00]	65.00 [59.00, 69.00]	0.985
BMI	21.50 [19.70, 23.70]	21.80 [19.50, 24.10]	21.20 [19.80, 23.40]	0.129
Chemotherapy cycles	5.00 [2.00, 8.00]	3.00 [1.00, 5.00]	7.00 [5.00, 11.00]	**<0.001**
Hospitalizations	7.00 [4.00, 12.00]	4.50 [2.00, 7.00]	10.00 [6.00, 15.30]	**<0.001**
WBC	6.90 [5.49, 9.17]	6.56 [5.19, 8.78]	7.07 [5.93, 9.55]	**<0.001**
RBC	3.77 [3.30, 4.15]	3.76 [3.28, 4.17]	3.78 [3.33, 4.15]	0.943
HGB	112.00 [99.90, 125.00]	112.00 [99.00, 125.00]	112.00 [101.00, 124.00]	0.603
PLT	209.00 [160.00, 258.00]	208.00 [161.00,268.00]	212.00 [160.00,241.00]	0.357
NEUT	72.10 [64.30, 79.20]	70.60 [63.10, 78.30]	74.10 [66.00, 79.60]	**<0.001**
LYM	17.30 [12.00, 23.30]	18.80 [12.80, 25.10]	16.10 [11.50, 21.60]	**<0.001**
MONO	7.30 [5.40, 9.48]	7.40 [5.50, 9.73]	7.11 [5.20, 9.10]	0.147
NLR	4.38 [2.83, 7.09]	3.74 [2.53, 6.10]	4.90 [3.33, 7.67]	**<0.001**
NMR	10.10 [7.11, 14.40]	9.34 [6.74, 13.20]	10.90 [7.57, 15.40]	**<0.001**
NPR	0.35 [0.27, 0.43]	0.33 [0.26, 0.42]	0.36 [0.29, 0.44]	**0.001**
IBIL	7.30 [5.70, 9.39]	7.00 [5.31, 9.42]	7.45 [6.10, 9.31]	**0.007**
ALT	18.00 [12.70, 26.30]	17.90 [13.00, 28.30]	18.20 [12.30, 24.90]	0.218
AST	23.40 [19.40, 29.40]	23.80 [18.90, 29.80]	23.20 [19.80, 28.70]	0.858
TBIL	9.89 [7.63, 12.60]	9.46 [7.22, 12.70]	10.10 [8.08, 12.30]	**0.013**
DBIL	2.40 [1.59, 3.40]	2.30 [1.50, 3.43]	2.50 [1.63, 3.38]	0.199
TP	66.80 [62.30, 69.90]	66.30 [61.70, 71.10]	66.90 [62.50, 69.20]	0.636
ALB	40.00 [36.50, 42.50]	39.90 [36.50, 42.70]	40.20 [36.80, 42.30]	0.764
GLB	26.30 [23.50, 29.40]	26.30 [22.30, 30.60]	26.30 [24.30, 28.60]	0.897
A/G	1.54 [1.31, 1.75]	1.52 [1.26, 1.81]	1.55 [1.34, 1.71]	0.943
Urea	5.89 [4.74, 7.56]	5.73 [4.58, 7.20]	6.05 [4.99, 7.64]	0.059
CREA	78.80 [66.00, 92.30]	76.60 [63.70, 91.90]	82.00 [68.60, 93.10]	**0.004**
UA	330.00 [278.00,394.00]	326.00 [265.00,398.00]	332.00[288.00,393.00]	0.180
CEA	3.69 [2.11, 9.74]	3.70 [2.05, 9.43]	3.68 [2.25, 9.81]	0.556
PE				<0.001
No	608 (75.20%)	353 (87.40%)	255 (63.10%)	
Yes	200 (24.80%)	51 (12.60%)	149 (36.90%)	
Gender				<0.001
Male	683 (84.50%)	315 (78.00%)	368 (91.10%)	
Female	125 (15.50%)	89 (22.00%)	36 (8.90%)	
Drink				<0.001
No	683 (84.50%)	377 (93.30%)	306 (75.70%)	
Yes	125 (15.50%)	27 (6.70%)	98 (24.30%)	
Smoke				<0.001
No	518 (64.10%)	322 (79.70%)	196 (48.50%)	
Yes	290 (35.90%)	82 (20.30%)	208 (51.5%)	
Diabetes				0.999
No	731 (90.50%)	366 (90.60%)	365 (90.30%)	
Yes	77 (9.50%)	38 (9.40%)	39 (9.70%)	
Hypertension				0.667
No	636 (78.70%)	321 (79.50%)	315 (78.00%)	
Yes	172 (21.30%)	83 (20.50%)	89 (22.00%)	
CHD				0.008
No	759 (93.90%)	370 (91.60%)	389 (96.30%)	
Yes	49 (6.10%)	34 (8.40%)	15 (3.70%)	
Stage				0.053
Stage I	29 (3.60%)	21 (5.20%)	8 (2.00%)	
Stage II	96 (11.90%)	46 (11.40%)	50 (12.40%)	
Stage III	330 (40.80%)	171 (42.30%)	159 (39.40%)	
Stage IV	353 (43.70%)	166 (41.10%)	187 (46.30%)	
Histology				0.005
Adenocarcinoma	327 (40.50%)	183 (45.30%)	144 (35.60%)	
Squamous	333 (41.20%)	151 (37.40%)	182 (45.00%)	
SCLC	135 (16.70%)	60 (14.90%)	75 (18.60%)	
Other lung cancers	13 (1.60%)	10 (2.50%)	1 (0.70%)	

Statistically significant differences are marked with bold font.

### 3.2 Predictor screening

**Figure 2:**
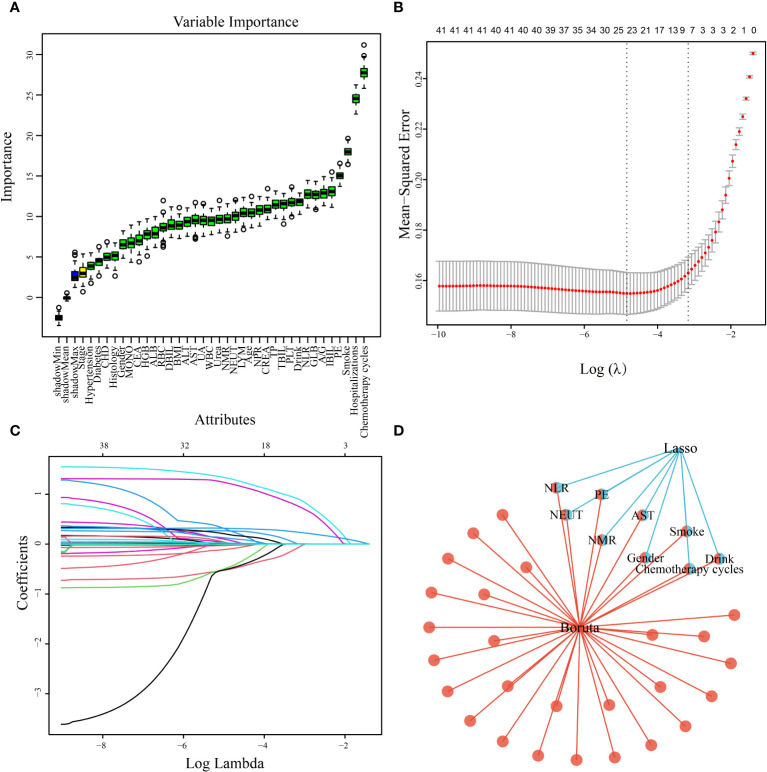
Predictor screening results. **(A)** Boruta; **(B)** Factor screening based on the LASSO regression model, with the left dashed line indicating the best lambda value for the evaluation metrics (lambda. min) and the right dashed line indicating the lambda value for the model where the evaluation metrics are in the range of the best value by one standard error (lambda.1se); **(C)** LASSO regression model screening variable trajectories; **(D)** common predictors between Boruta and LASSO.

A total of 808 patients undergoing chemotherapy for lung cancer after data imbalance were divided into a training group consisting of 565 patients and a test group consisting of 243 patients, following a ratio of 7:3. Statistical analysis revealed no significant differences between the two groups (**Table 3**). Utilizing the Boruta algorithm, an extension of the RF algorithm, enabled the identification of the actual feature set by accurately estimating the importance of each feature. The Boruta algorithm identified 35 key factors, including Drink, Smoke, Chemotherapy cycles, Hospitalizations, PE, NEUT, LYM, MONO, NLR, and NMR, etc (**Figure 2A**). In contrast, LASSO regression serves as a compression estimation method that accomplishes variable selection and complexity adjustment through the formulation of an optimization objective function incorporating penalty terms. In this study, LASSO regression was utilized to identify characteristic factors such as Gender, Drink, Smoke, Chemotherapy cycles, PE, NEUT, NLR, NMR, and AST (**Figures 2B, C**). Through a comparative analysis of the outcomes obtained from LASSO regression and Boruta algorithm screening, we identified a common subset of feature variables selected by both methods. These selected features were ultimately utilized in the construction of the model and consisted of Gender, Drink, Smoke, Chemotherapy cycles, PE, NEUT, AST, NLR, and NMR (**Figure 2D**).

**Table 3:** Training set and Test set variability analysis.

Variable	Total (N = 808)	Train set (N = 565)	Test set (N = 243)	*P*
Age	65.00 [59.00, 70.00]	65.00 [59.00, 70.00]	65.00 [58.00, 70.00]	0.727
BMI	21.50 [19.7, 23.70]	21.50 [19.60, 23.70]	21.60 [20.00, 23.60]	0.497
Chemotherapy cycles	5.00 [2.00, 8.00]	5.00 [2.00, 8.00]	5.00 [2.00, 9.00]	0.737
Hospitalizations	7.00[4.00, 12.00]	7.00 [4.00, 12.00]	7.00 [3.00, 12.00]	0.927
WBC	6.90 [5.49, 9.17]	6.82 [5.49, 8.94]	7.37 [5.47, 9.89]	0.075
RBC	3.77 [3.30, 4.15]	3.77 [3.33, 4.14]	3.76 [3.29, 4.19]	0.644
HGB	112.00 [99.90, 125.00]	112.00 [99.30, 124.00]	112.00 [100.00, 125.00]	0.810
PLT	209.00 [160.00, 258.00]	210.00 [159.00, 262.00]	209.00 [163.00, 243.00]	0.411
NEUT	72.10 [64.30, 79.20]	71.60 [63.60, 78.90]	73.30 [66.10, 80.10]	0.073
LYM	17.30 [12.00, 23.30]	17.50 [12.20, 23.70]	16.80 [11.40, 23.00]	0.249
MONO	7.30 [5.40, 9.48]	7.23 [5.50, 9.50]	7.40 [5.05, 9.30]	0.456
NLR	4.38 [2.83, 7.09]	4.27 [2.73, 6.89]	4.46 [2.97, 7.60]	0.137
NMR	10.10 [6.79, 14.03]	10.20 [7.00, 14.40]	10.00 [7.46, 14.50]	0.375
NPR	0.35 [0.27, 0.43]	0.34 [0.27, 0.43]	0.36 [0.28, 0.44]	0.108
IBIL	7.30 [5.70, 9.39]	7.24 [5.79, 9.30]	7.40 [5.60, 9.72]	0.700
ALT	18.00 [12.70, 26.30]	18.20 [12.40, 27.00]	17.40 [13.30, 24.30]	0.853
AST	23.40 [19.40, 29.40]	23.40 [19.30, 29.00]	23.50 [19.70, 29.70]	0.805
TBIL	9.89 [7.63, 12.60]	9.80 [7.70, 12.40]	9.90 [7.50, 12.80]	0.955
DBIL	2.40 [1.59, 3.40]	2.44 [1.60, 3.40]	2.30 [1.48, 3.39]	0.467
TP	66.80 [62.30, 69.90]	66.70 [62.20, 70.00]	67.20 [62.40, 69.80]	0.939
ALB	40.00 [36.50, 42.50]	39.90 [36.70, 42.40]	40.20 [36.30, 42.70]	0.931
GLB	26.30 [23.50, 29.40]	26.30 [23.70, 29.10]	26.40 [22.80, 30.00]	0.967
A/G	1.54 [1.31, 1.75]	1.54 [1.32, 1.75]	1.55 [1.26, 1.77]	0.975
Urea	5.89 [4.74, 7.56]	5.92 [4.69, 7.63]	5.84 [4.89, 7.32]	0.540
CREA	78.80 [66.00, 92.30]	78.50 [66.60, 92.20]	79.80 [64.00, 92.90]	0.889
UA	330.00 [278.00, 394.00]	330.00 [279.00, 394.00]	330.00 [277.00, 395.00]	0.951
CEA	3.69 [2.11, 9.74]	3.69 [2.14, 9.87]	3.66 [2.03, 8.29]	0.447
Gender, n (%)				0.298
Male	683 (84.50%)	483 (85.50%)	200 (82.30%)	
Female	125 (15.50%)	82 (14.50%)	43 (17.70%)	
Drink, n (%)				0.385
No	683 (84.50%)	473 (83.70%)	210 (86.40%)	
Yes	125 (15.50%)	92 (16.30%)	33 (13.60%)	
Smoke, n (%)				0.087
No	518 (64.10%)	351 (62.10%)	167 (68.70%)	
Yes	290 (35.90%)	214 (37.90%)	76 (31.30%)	
Diabetes, n (%)				0.864
No	731 (90.50%)	510 (90.30%)	221 (90.90%)	
Yes	77 (9.50%)	55 (9.70%)	22 (9.10%)	
Hypertension, n (%)				0.371
No	636 (78.70%)	450 (79.60%)	186 (76.50%)	
Yes	172 (21.30%)	115 (20.40%)	57 (23.50%)	
CHD, n (%)				0.226
No	759 (93.90%)	535 (94.70%)	224 (92.20%)	
Yes	49 (6.10%)	30 (5.30%)	19 (7.80%)	
Stage, n (%)				0.779
Stage I	29 (3.60%)	20 (3.50%)	9 (3.70%)	
Stage II	96 (11.90%)	71 (12.60%)	25 (10.30%)	
Stage III	330 (40.80%)	232 (41.10%)	98 (40.30%)	
Stage IV	353 (43.70%)	242 (42.80%)	111 (45.70%)	
Histology, n (%)				0.537
Adenocarcinoma	327 (40.50%)	221 (39.10%)	106 (43.60%)	
Squamous	333 (41.20%)	241 (42.70%)	92 (37.90%)	
SCLC	135 (16.70%)	93 (16.50%)	42 (17.30%)	
Other lung cancers	13 (1.60%)	10 (1.80%)	3 (1.20%)	
PE				0.237
No	608 (75.20%)	418 (74.00%)	190 (78.20%)	
Yes	200 (24.80%)	147 (26.00%)	53 (21.80%)	

### 3.3 Model performance

**Figure 3:**
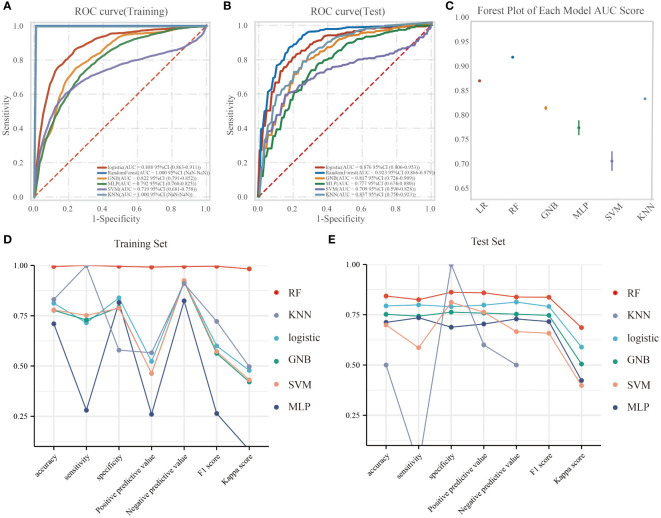
The performance and comparison of six different predictive models. **(A)** The training set ROC curve; **(B)** The test set ROC curve; **(C)** Forest plot of AUC values; **(D)** Evaluation metrics for the training set; **(E)** Evaluation metrics for the test set.

In the training dataset, the RF model exhibited superior predictive performance with an AUC of 1.00, indicating a high level of accuracy in prediction. In contrast, the AUC values for the remaining five models were as follows: 0.888, 95%CI(0.863-0.911) for LR, 0.822, 95%CI(0.791-0.852) for GNB, 0.792, 95%CI(0.760-0.825) for MLP, 0.719, 95%CI(0.681-0.758) for SVM, and 1.000, 95%CI(NaN- NaN) for KNN (**Figure 3A**). The F1 scores for these models were as follows: LR 0.816, RF 0.998, GNB 0.756, MLP 0.736, SVM 0.679, and KNN nan. In the test set, the AUC values for LR, RF, GNB, MLP, SVM, and KNN were 0.876(95%CI(0.806-0.953)), 0.923(95%CI(0.866-0.979)), 0.817(95%CI(0.726-0.909)), 0.777(95%CI(0.674-0.880)), 0.709(95%CI(0.590-0.828)), and 0.837(95%CI(0.750-0.923)), respectively (**Figure 3B**). The corresponding F1 scores were 0.791, 0.837, 0.747, 0.716, 0.658, and nan for LR, RF, GNB, MLP, SVM, and KNN, respectively. The forest plot comparing the AUC scores of the six ML models is presented in **Figure 3C**. In this study, the accuracy, sensitivity, specificity, positive predictive value, negative predictive value, and kappa value of each model were computed and compared (**Figures 3D, E**). While the RF model exhibited exceptional performance on the training set, the Logistic Regression model was ultimately selected as the optimal model due to concerns regarding potential overfitting.

### 3.4 The logistic regression model

The results of the univariate logistic analysis are summarized in **Supplementary Table 1**. 12 variables were statistically significant: Gender, Drink, Smoke, CHD, Chemotherapy cycles, Hospitalizations, PE, NEUT, LYM, NLR, NMR, and CEA. **Table 4** presents the coefficients and odds ratios (OR) for the nine predictor variables included in the model. The logistic equation was as follows:

**Table 4:** Risk factors and their parameters of the logistic model.

Variables	Coefficients	OR(95%CI)	p
Intercept	-2.954	0.052(0.006-0.395)	**0.006**
Gender	-0.424	0.655(0.321-1.297)	0.233
Drink	-0.049	0.952(0.464-1.963)	0.893
Smoke	1.754	5.776(3.292-10.375)	**<0.001**
Chemotherapy cycles	0.395	1.484(1.380-1.606)	**<0.001**
PE	1.417	4.123(2.389-7.274)	**<0.001**
NLR	0.083	1.087(1.007-1.174)	**0.034**
NMR	0.017	1.018(0.998-1.038)	0.077
AST	0.009	1.009(1.003-1.019)	**0.020**
NEUT	-0.008	0.992(0.962-1.026)	0.639

OR, odds ratio; CI, confidence interval.

**Figure 4:**
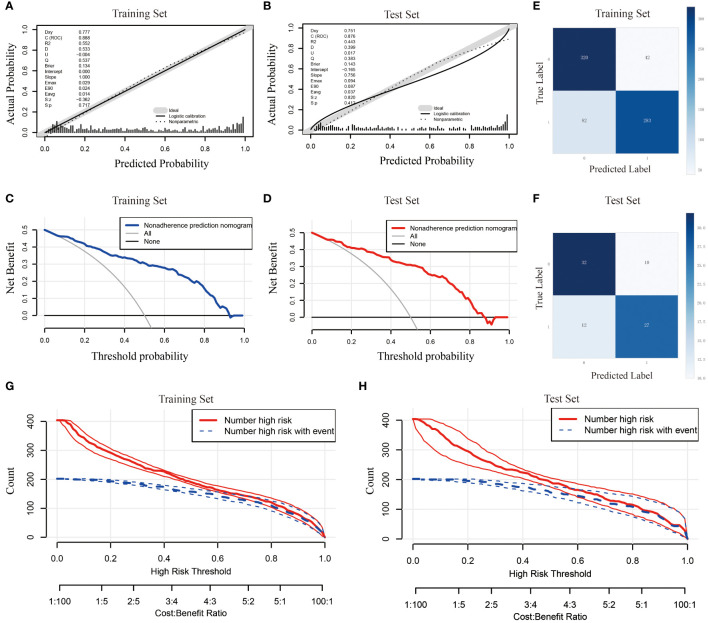
Comprehensive evaluation of the logistic regression model. **(A)** Calibration curve for the training set; **(B)** Calibration curve for the test set; **(C)** Decision curve analysis for the training set; **(D)** Decision curve analysis for the test set; **(E)** Confounding matrix for the training set; **(F)** Confounding matrix for the test set; **(G)** Clinical impact curve for the training set; **(H)** Clinical impact curve for the test set.

Statistically significant differences are marked with bold font.

y = - 2.954 - 0.424×Gender - 0.049×Drink + 1.754×Smoke + 0.395×Chemotherapy cycles + 1.417×PE + 0.083×NLR + 0.017×NMR + 0.009×AST - 0.008×NEUT. In this study, we evaluated the prediction accuracy and calibration of the model by calibration curve analysis of the training and test sets. The calibration curve results showed that the model in the training set had high prediction accuracy with a Somers’ D coefficient of 0.777 and an area under the ROC curve of 0.888, indicating that the model had excellent discriminative ability (**Figure 4A**). In addition, the logistic regression calibration slope of the training set model was close to the ideal value of 1.000, with an intercept of 0.000, showing excellent calibration. The Brier score of 0.134 reflected the high reliability of the model predictions. In contrast, the model in the test set maintained a high discriminative power with an area under the ROC curve of 0.876, although there was a slight decrease in prediction accuracy (Somers’ D coefficient of 0.751) (**Figure 4B**). The decision curve for the training set (**Figure 4C**) shows that the model provides significantly higher net gains than the baseline strategy when the threshold probabilities are between 0.1 and 0.9. On the test set (**Figure 4D**), the model similarly demonstrates good net returns, especially in the range of threshold probabilities from 0.1 to 0.85, where it maintains a high level of net returns. The confusion matrix results show the difference in the model’s performance on different datasets. In the training set (**Figure 4E**), the model correctly identified 320 true negatives and 283 true positives, and misidentified 42 false positives and 82 false negatives, with a true positive rate (sensitivity) of 77.5% and a true negative rate (specificity) of 88.4%. In the test set (**Figure 4F**), the model correctly identified 32 true negatives and 27 true positives and misidentified 10 false positives and 12 false negatives, for a true positive rate of 69.2% and a true negative rate of 76.2%. Finally, we plotted clinical impact curves (CICs) to assess the net gain in clinical utility and applicability of the model with the highest diagnostic value. The clinical impact curves (**Figures 4G, H**) provide information on the ability of the models to predict high-risk patients at different cost-benefit ratio thresholds. The curves for both the training and test sets show that when the threshold probability is greater than the 65% predictive score probability value, the predictive model’s determination of those at high risk of developing an infection in the lungs after chemotherapy is highly matched to those who actually develop an infection, confirming that the predictive model is clinically highly effective.

### 3.5 SHAP-based model interpretability analysis

**Figure 5:**
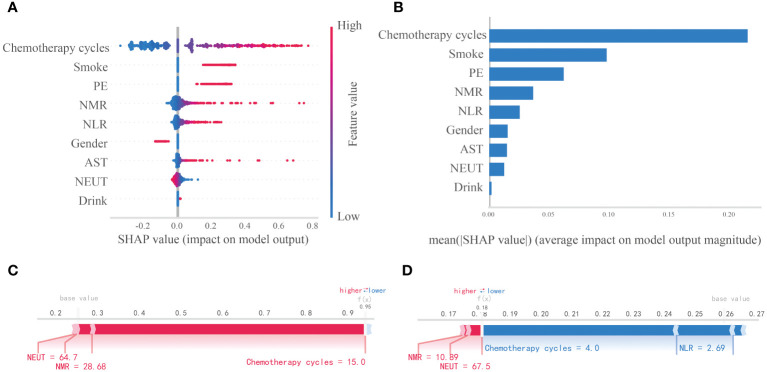
Interpretability analysis of logistic regression models. **(A)** SHAP dendrogram of features of the logistic regression model. **(B)** Importance ranking plot of features of the logistic regression model. **(C, D)** Interpretability analysis of 2 independent samples.

This study assessed the relative significance of various factors influencing the susceptibility to lung infections following chemotherapy in patients with lung cancer. **Figure 5A** visually represents this ranking, with each point denoting a sample and the color gradient from blue to red indicating the magnitude of the sample eigenvalues. The vertical axis displays the importance ranking of features, along with the correlation and distribution of each eigenvalue with the SHAP value. The impact of the top nine features in the importance ranking on prediction outcomes is illustrated in **Figure 5B**. Specifically, Chemotherapy cycles, Smoke, and PE exhibit positive contributions to the predictive results, while NEUT demonstrate negative influences on the model’s output. **Figure 5B** illustrates the hierarchical significance of features in the logistic regression model. The vertical axis displays individual features in descending order of importance, while the horizontal axis represents average SHAP values. The analysis reveals that Chemotherapy cycles, Smoke, PE, NMR, and NLR are the top five features ranked by importance, indicating their critical influence on the presence of a lung infection. To enhance comprehension of the model’s decision-making process at the individual level, we conducted a detailed interpretability analysis on two representative samples, as illustrated in **Figures 5C, D**. By visualizing the SHAP values of these samples, we could discern the impact of each feature on the model’s predictions for these specific instances.

### 3.6 Construction of nomograms

**Figure 6:**
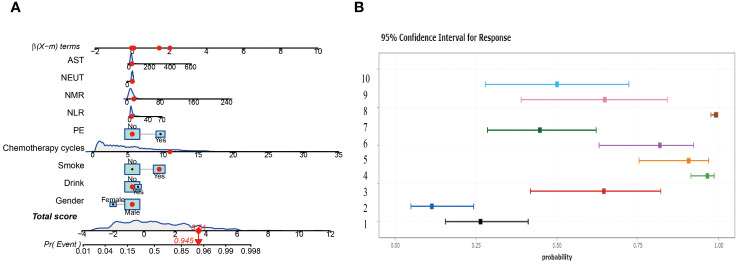
Construct two different nomograms. **(A)** Interactive Nomogram. **(B)** Dynamic Nomogram showing risk profiles for ten scenarios.

In this study, two nomograms were constructed, integrating nine important predictor variables such as alcohol consumption, smoking, and chemotherapy cycle to visually assess the risk of lung infection after chemotherapy. **Figure 6A** shows an interactive nomogram with a score of 3.51 for the example patient, corresponding to a 94.5% probability of infection, providing a quick and easy-to-interpret risk assessment. **Figure 6B** illustrates a dynamic nomogram with different risk profiles derived from 10 combinations of variables.

## 4 Discussion

This research investigated the predictive factors associated with post-chemotherapy lung infection in patients with lung cancer and developed a logistic regression-based predictive model that effectively estimates the likelihood of lung infection following chemotherapy. By employing meticulous feature selection and conducting multi-model comparative validation, this study highlights the significance of various key predictors and offers a valuable tool to aid in clinical decision-making.

Zhou D et al. conducted a retrospective analysis of 244 non-small cell lung cancer (NSCLC) patients who underwent surgical interventions from June 2015 to January 2017. Through applying LASSO regression and logistic regression analyses, the researchers identified independent risk factors for postoperative pulmonary infection (PPI) in NSCLC patients and subsequently developed a predictive model based on these findings (26). Jong-Ho Kim and colleagues pioneered the application of ML techniques for the prognostication of postoperative pulmonary complications (PPCs), employing a suite of five algorithms, namely LR, random forests (RFs), light-gradient boosting machines (LightGBM), extreme-gradient boosting machines (XGBoost) and MLP for the construction and assessment of predictive models (27). Xue et al. established a predictive model utilizing preoperative and intraoperative data to detect the likelihood of postoperative pneumonia. Their research delved into the application of machine learning in predicting a range of postoperative complications, including pneumonia, within the context of PPCs. Nevertheless, the authors failed to emphasize unique characteristics and risk factors beyond pneumonia linked to PPCs, potentially diverting attention away from PPCs (28). While predictive models have been created for complications in lung cancer patients, there is a scarcity of predictive models utilizing ML algorithms for assessing the risk of lung infection following chemotherapy for lung cancer.

The dysregulation of the autoimmune system, exacerbated by chemotherapy-induced immune cell depletion, tumor cell infiltration, impaired antibody-complement generation, and dysregulation of the inflammatory system, disrupts immune homeostasis and heightens susceptibility to concurrent lung infections (29–31). This risk is further compounded in individuals with comorbidities such as chronic bronchitis, chronic obstructive pulmonary disease, interstitial lung disease, pulmonary atelectasis, and other organic diseases (32, 33). The occurrence of lung infection during chemotherapy is a prevalent and challenging complication that hinders the efficacy of treatment and exacerbates the health status of patients, ultimately impacting their prognosis and increasing the financial burden of medical care. As such, our research holds significant clinical importance in examining the determinants of lung infection during chemotherapy and implementing timely and efficient interventions for patients with lung cancer.

This study employed a dual methodology of Boruta’s algorithm and LASSO regression to identify predictors for accurate feature selection and model stability. The selected features encompassed variables such as alcohol consumption status, smoking habits, chemotherapy cycles, hospitalization frequency, presence of lung pleural fluid, neutrophil count, AST, NLR, and NMR, all of which have demonstrated significant correlations with the prognosis of lung cancer patients in prior research. Wei Guo et al. colleagues created a predictive model utilizing artificial neural network (ANN) technology to forecast infection rates in lung cancer patients undergoing chemotherapy (34). The researchers employed a logistic regression (LR) model to analyze the data and identify statistically significant variables. Their results indicated a positive correlation between length of hospital stay and infection risk, which aligns with our research findings. However, the researchers discovered that a prior diagnosis of diabetes was linked to an increased likelihood of lung infection, a finding that did not align with our results. This discrepancy may be attributed to the limited sample size of the previous study, which only included 80 cases. Zhouzhou Ding et al. explored the risk factors for PPI in patients with non-small cell lung cancer (NSCLC), developed a risk model, and conducted predictive modeling for PPI. Their research revealed that the chemotherapy cycle, identified as an independent risk factor, had a notable impact on the occurrence of PPI (26). This is in general agreement with our findings. Our findings emphasize the importance of monitoring and managing these factors during chemotherapy management.

After comparing these models, it is observed that while the RF model exhibits superior performance in the training set, its propensity for overfitting necessitates the selection of the logistic regression model as the optimal choice due to its strong generalization capabilities in the external test set. Logistic regression models are favored for their predictive accuracy and interpretability, which are essential qualities for practical clinical implementation. The importance of constructing disease prediction models lies in identifying high-risk patients and mitigating the risk for individuals who may fall into the high-risk category, thereby benefiting patients overall. Consequently, the clinical interpretability of ML models holds significant value in medical practice. In this research, we utilized the SHAP method to provide both global and local interpretations of the ML model, enhancing its visual representation and transparency. Kaidi Gong et al. have observed that the SHAP method exhibits superior consistency and performance compared to conventional weight-based interpretation methods, and the SHAP algorithm demonstrates greater stability across various models. In contrast to the Local Interpretable Model-agnostic Explanations (LIME) method, SHAP demonstrates strong performance in both global and individual interpretation tasks, while LIME shows less consistency in individual analysis (35). Yasunobu Nohara and colleagues further substantiated that SHAP values exhibit superior interpretability compared to the coefficients of generalized linear regression models, as evidenced through a comparative analysis of interpretation outcomes with other established methodologies. Additionally, they found that SHAP summary plots offer more effective visualization of results than feature importance plots (36). The utilization of SHAP value analysis in this research offers a novel lens through which to comprehend the model’s decision-making process. Through this method, we were able to elucidate the specific contributions of individual predictors to the model’s decision-making, ultimately improving the transparency and interpretability of the model. Notably, factors such as chemotherapy cycle, smoking, PE, and NMR were underscored for their significance, consistent with prior research findings and reaffirmed their pivotal role in predicting post-chemotherapy lung infections.

Despite the results of this study, there are some limitations. Firstly, being a retrospective study, there is a potential for omitted data and selection bias to impact the results. Secondly, the small sample size of this study and the fact that the sample was collected from a single center may limit the generalizability of the findings. The potential incorporation of prospective design and multicenter data in future studies, coupled with integrating additional patient data and utilizing advanced machine learning techniques, is anticipated to enhance model performance. This improvement aims to validate the robustness and generalizability of the model, ultimately leading to the development of more personalized and precise treatment management strategies for patients with lung cancer.

## 5 Conclusion

This study has effectively developed a predictive tool utilizing logistic regression modeling to forecast lung infections following chemotherapy in lung cancer patients. The tool demonstrates high predictive accuracy and holds substantial clinical relevance. By identifying and assessing crucial predictors, this research establishes a valuable scientific foundation for the prevention and treatment of post-chemotherapy complications in lung cancer patients, ultimately enhancing patient survival quality and prognostic outcomes. Future work will focus on further validating the model’s validity and exploring integrating these predictive tools into clinical practice to improve the prediction of treatment consequences in lung cancer patients.

## Data Availability

The original contributions presented in the study are included in the article/**Supplementary Material**. Further inquiries can be directed to the corresponding author.
